# Sleep and PTSD in the Military Forces: A Reciprocal Relationship and a Psychiatric Approach

**DOI:** 10.3390/brainsci11101310

**Published:** 2021-10-01

**Authors:** Emeric Saguin, Danielle Gomez-Merino, Fabien Sauvet, Damien Leger, Mounir Chennaoui

**Affiliations:** 1 Psychiatric Department, Begin Military Teaching Hospital, 94160 Saint-Mandé, France; 2 VIFASOM (Vigilance Fatigue Sommeil et Santé Publique) EA 7330, Université de Paris, 75005 Paris, France; 3 French Armed Forces Biomedical Research Institute, 91220 Brétigny-sur-Orge, France; dangomez51@gmail.com (D.G.-M.); fabien.sauvet@gmail.com (F.S.); mounirchennaoui@gmail.com (M.C.); 4 Centre du Sommeil et de la Vigilance, Hôtel-Dieu, APHP, 75004 Paris, France; damien.leger@aphp.fr

**Keywords:** sleep, combat-related trauma, nightmares, PTSD, military, REM sleep

## Abstract

Sleep disturbances are well-recognised symptoms of Post-Traumatic Stress Disorder (PTSD). This review updates knowledge regarding the relationship between sleep during deployment, combat-related trauma, and PTSD in military personnel, from which the importance of restorative sleep results. The description of the characteristics of sleep in military forces with the considerable roles of the operational and training contexts highlights the important consequences of degraded sleep. Indeed, a lot of data suggest a dynamic link between sleep and the onset and chronicity of PTSD. We propose a reciprocal relationship model with strategies strongly recommended or already adopted by the military to promote restorative sleep before and after combat exposure. Among the alterations in a variety of sleep architecture and sleep patterns described in PTSD, the physiological hypothesis of REM sleep fragmentation in the development of PTSD symptoms may be important because REM sleep is generally associated with emotional memory. Finally, we address clinical and research perspectives that could be used to detect or restore sleep continuity before and during military deployment to possibly alleviate nightmares and insomnia related to combat exposure and PTSD occurrence and improve our understanding of sleep in PTSD.

## 1. Introduction

In our time, Post-Traumatic Stress Disorder (PTSD) is associated with traumatic events such as accidents, assaults and terrorist attacks, and more specifically in the military in relation to combat-related trauma [1,2]. It has been argued that sleep disturbances play a critical role in the maintenance of PTSD and are a hallmark of the disorder [3,4,5].

Sleep is a basic physiological need that is crucial for human life and serves multiple restorative functions in the body and the brain. Indeed, sleep improves memory recall, regulates metabolism, and reduces mental fatigue, while also playing important roles in tissue repair, synaptic homeostasis, and immune-inflammatory control (reviewed by [6] and [7]). Adequate sleep duration and quality are vital for optimal mental and physical health. Healthy sleep should be of sufficient duration and good quality, of appropriate timing and regularity, and should be free of sleep disorders or disturbances. Many studies in different countries have shown that, despite a regular average total sleep time in the adult population of around 7 h, sleep debt is frequently seen in professionally active populations (e.g., health care professions, drivers, soldiers, police officers, etc.). Based on characteristics of electroencephalography (EEG) and electromyography (EMG) signals, sleep wake states can be classified as waking, rapid eye movement (REM), and non-rapid eye movement (NREM) sleep, also known as slow-wave sleep (SWS).

Military operational personnel are often exposed to significant sleep debt associated with climatic, nutritional, psychological, mental and physical stressors and/or bad sleep conditions [8]. Furthermore, even when service members do have opportunities for sleep, several additional environmental disruptors in the military operational context make it difficult to obtain restorative sleep, examples of these being light, noise, temperature, and air pollution [9]. These operational conditions often make it impossible to achieve the 7–8 h of continuous nighttime sleep duration recommended by the Sleep Research Society and the American Academy of Sleep Medicine [10].

The present review focused on the interrelationship between sleep disturbances and PTSD in the military population. This population represents a relatively homogeneous population in age which is exposed to well-defined physical and psychological constraints and to life-threatening occupational events, and whose medical state of health is rigorously monitored over the long-term. All of this facilitates clinical and fundamental research on sleep parameters that predict and/or aggravate chronic PTSD. The other professional settings (health professions, drivers, police officers) that we cite as examples present similarities (e.g., professional exposure to physical and psychological stressors and sleep debt) placing them at risk of PTSD, and are therefore of interest to the reciprocal relationship model. That’s why the study of the interrelationship between altered sleep and chronic PTSD in military forces could open up unique perspectives for biomedical research and care.

## 2. Sleep in the Military Forces: Epidemiology and Recommendations

### 2.1. Sleep Schedules, Quantity and Quality

Sleep problems are prevalent among US army service members who have been deployed to combat operations in Iraq and Afghanistan, and sleep disturbances persist for months or even years after deployments have ended [8,11]. In US soldiers of a brigade combat team that was deployed to Iraq for periods between 6–15 months, the three components of good sleeping (timing, duration, and quality) were challenged, and their self-reported sleep duration ranged from 5.8 to 6.5 h per night, regardless of deployment status, fatigue, depression, posttraumatic stress disorder, or pain syndromes [12]. At the same time, the US Millennium Cohort study survey of >55,000 men and women service members, from all military service branches deployed to Iraq and Afghanistan, described a significant association between the deployment status with shorter self-reported average sleep duration and increased trouble sleeping [13]. US Navy personnel in the Afghanistan combat theatre self-reported an average of 5.9 h of sleep per day despite needing an average of 6.8 h to feel well rested, and fifty-seven percent reported insufficient sleep interacting with mission type [14]. Additionally, those who said they slept less were more likely to report causing an accident or making an error that affected the mission. In US military cadets followed for sleep/wake patterns during four years using wrist activity monitors, significant and chronic sleep deprivation was found to average over 3 h per day for each day [15]. In European armies, there are less data on sleep during military deployments. However, it was shown using sleep questionnaires on German soldiers deployed in Afghanistan that sleep quality and daytime sleepiness were already impaired during the predeployment training phase and remained at this level during the deployment phase [16]. In U.K. soldiers deployed in Afghanistan, longer deployment time was associated with greater sleep problems within 4–6 months post-deployment [17]. Finally, there are no data on the prevalence of sleep problems among French soldiers during their deployment to a combat zone (i.e., the operational environment). However, data on a large, real world French-American study conducted in the 2000s (known as “Operation Pegasus”) on twenty-seven healthy volunteers from a US Air Force Reserve Unit after an eastbound flight across seven time zones, showed that SWS and REM sleep debt increased during the flight and on the first day of recovery, but was fully recovered after the following three nights [18]. A more recent study on French submariners working during a 70-day mission on a schedule of a 24-h/day, three consecutive day shift showed no significant effects on sleep, sleepiness and confusion, likely because of the protective influence of the organised regular shifts [19]. In addition, prospective studies conducted on military personnel during the systematic annual medical check-ups or in their workplace that involve atypical schedules have shown a high prevalence of hypersomnia and daytime hypersomnia with strong daytime repercussions [20,21,22].

There is now a consensus on the consequences of repeated sleep debt in soldiers: the ability to recover from the mentally and physically demanding tasks inherent in military operations decreases, and military performance may be degraded, compromising personal safety and resources ([23], reviewed in [8]). The impact of deployment on neurocognitive functioning, sleepiness, and mood in healthy, non-concussed US service members of Marine Corps units has been recently investigated [24]. Significant differences were found compared to normal values, and negative mood states were found to have significant negative relationships with several domains of neurocognitive performance, while measures of positive mood states and sleepiness did not. Furthermore, recent data have shown that poor sleep quality slightly increased the risk of committing military-specific high-risk behaviors in four diverse U.S. Army samples of 2296 people aged approximately 25 years, and that a longer duration reduced the risk to a greater extent, even when controlling for a number of relevant demographic factors [25]. Longer sleep duration also predicted decreased high-risk behaviors. Finally, a recent study of Army special operations forces soldiers, primarily men, described that those who slept for less than four hours were 2.35 times more likely to have a musculoskeletal injury than those who slept for more than eight hours, after adjusting for other covariates [26].

### 2.2. Sleep Disorders

With respect to sleep disorders in the military, the most frequently diagnosed and often subsequently related to combat zone deployments are insomnia and obstructive sleep apnea (OSA) [27,28,29], reviewed by [11,30]. In the US military population, insomnia was recently shown to be an important risk factor for motor vehicle accident-related injuries [31]. Sleep disorders have been particularly evidenced in active duty service members and military Veterans pre- and post-deployment and across sexes [32,33], using self-reported data and polysomnography. In the US Millennium Cohort Study survey, the pre-deployment insomnia diagnosis has been found to be associated with a significant increased risk for new-onset post-traumatic stress disorder (PTSD), depression, and anxiety positive screens following deployment, independently of other potential risk factors [34]. In this cohort, the presence of insomnia symptoms was also significantly associated with lower self-rated health, more lost work days, lower odds of deployment, higher odds of early discharge from military service, and more health care utilization [35]. A recent study also demonstrated that pre-deployment insomnia is associated with post-deployment PTSD and suicidal ideation in US Army soldiers [36]. The insomnia diagnosis in US Navy and Marine Corps personnel deployed to combat zones has been recently found to be associated with military occupation, with the highest rates among health care, law enforcement, and motor transport personnel [37]. This result shows the impact on sleep of irregular work schedules and of tasks performed outside normal working hours. Individuals then sometimes develop compensatory behaviors, such as excessive consumption of caffeine, energy drinks, and alcohol, which can perpetuate the problem and lead to chronic insomnia [30]. Consequently, in the manner of a vicious circle, insomnia can exacerbate the deployment experience and is a risk factor for traumatic stress reactions such as PTSD [30].

## 3. PTSD in the Military Forces

### 3.1. Clinical Specific Aspects and Prevalence

Post-Traumatic Stress Disorder (PTSD) is a complex psychiatric disorder that occurs following exposure to one or more traumatic events. The four main symptoms of PTSD are intrusion symptoms (e.g., unwanted upsetting memories, nightmares, flashbacks), avoidance (of trauma-related stimuli), increased arousal and reactivity (e.g., irritability, hypervigilance, sleep disturbance), and negative alteration of cognition and mood. It should also be noted that, in this definition, symptoms are characterized by their chronicity (lasting more than one month), their link with a traumatic event (symptoms are not due to medication, substance use or other illness) and their clinical significance (distress or functional impairment) [1].

The military population is particularly exposed to life-threatening events due to deployment in war zones. There may be a specificity in the study of PTSD in veterans because studies conducted in the civilian population find a majority of PTSD in female victims of sexual assault, whereas in the military, PTSD more often begins after a direct encounter with death [2]. Focusing on events involved in the onset of PTSD in French soldiers between 2010 and 2013, the French Armed Forces Epidemiologic and Public Health Centre (CESPA) identified that patients were confronted with death (thinking about dying themselves or seeing dead people) in 88.1% of cases [38]. These differences in traumatic experiences could explain the variations in symptomatology between military and civilian patients. Indeed, intrusive symptoms (e.g., replicative posttraumatic nightmares) are proportionately more prevalent in the veteran population (65%) than in the civilian trauma survivor population (20–30%) [39,40].

The epidemiology of PTSD is difficult to estimate because of population differences and wide variations in medical screening and diagnostic approaches between countries. In Europe, the estimated lifetime prevalence of PTSD among the civilian population was 1.9% (2.9% for women and 0.9% for men) [41]. This estimation could be surprizing given the more important prevalence of PTSD in the US: 6.8% for lifetime prevalence and 3.5% for past year prevalence [42]. This reflects conceptual differences between Europe and the US In the US, the definition of a traumatic event and, by extension, the definition of PTSD, is broader. According to the DSM IV, the criteria for PTSD include many common and specific symptoms of psychiatric disorders and the definition of traumatic experience, which is only related to a stressor, remains unclear [43]. In Europe, there is an important psychodynamic heritage in psychiatry which supports a distinction between the traumatic event and PTSD. In our view, PTSD is only the most extreme mode of reaction after a traumatic event, but there are a multitude of other possible “responses” (pathological or not) after a trauma, ranging from discrete changes to complete disorders (with all the criteria). As we will highlight in this study, sleep may be a key phenomenon that explains some of these differences in clinical outcomes after a traumatic event.

In France, two studies have found, respectively, a PTSD prevalence of 1.7% and 4.8% in army forces [44,45]. This prevalence estimation should be considered with caution due to important differences between military units. Nevertheless, there are logically more cases in the military than in the civilian population, making PTSD a major cause of operational disability.

This is of particular concern to military physicians in light of the poor efficacy of the pharmacological approach to PTSD and the risk of multiple trauma exposures for military personnel. In general, the definition of treatment response for PTSD is a 30% or greater decrease on the Clinician-Administered PTSD scale (CAPS) [46]. Despite this moderate expectation, the efficacy of selective serotonin reuptake inhibitors (SSRIs) (recommended pharmacotherapy in PTSD) is estimated to be zero [47] or better than placebo in only 20% of cases [48]. A recent meta-analysis showed, for all the pharmacotherapies used in PTSD, an effectiveness with an effect size that remains very moderate [49]. From a long term perspective, only 20–30% of the patients achieve complete remission [50] and there is residual symptomatology in about 1/3 of PTSD cases 10 years after the traumatic event [51]. Combat veterans with PTSD appear particularly resistant to pharmacotherapy [52]. Psychotherapy appears to be more effective. The US Department of Veterans Affairs (https://www.ptsd.va.gov/) (accessed on 23 August 2021) and the « PTSD: National Center for PTSD » page give that trauma-focused psychotherapies with the strongest evidence are Prolonged Exposure (PE), Cognitive Processing Therapy (CPT), and Eye Movement Desensitization and Reprocessing (EMDR). However, they also underline that veterans respond less to pharmacotherapy and psychotherapy treatments than active military personnel (the effect-size is lower).

The consequences of PTSD are therefore important both on a personal level (perceived suffering, inability to work, numerous comorbidities) and on a socio-familial level (significant impact on the family and colleagues). Other therapeutic options are needed to improve care and prevent the disease from becoming chronic.

### 3.2. Sleep and PTSD in Military Forces

#### 3.2.1. Sleep Characteristics in Military PTSD

Sleep disturbances (including trouble falling asleep, waking up repeatedly, trouble staying asleep, non-restorative sleep) occur for 74% of veterans with PTSD, which make it the primary medical complaint for this population [4,5].

**Figure 1:**
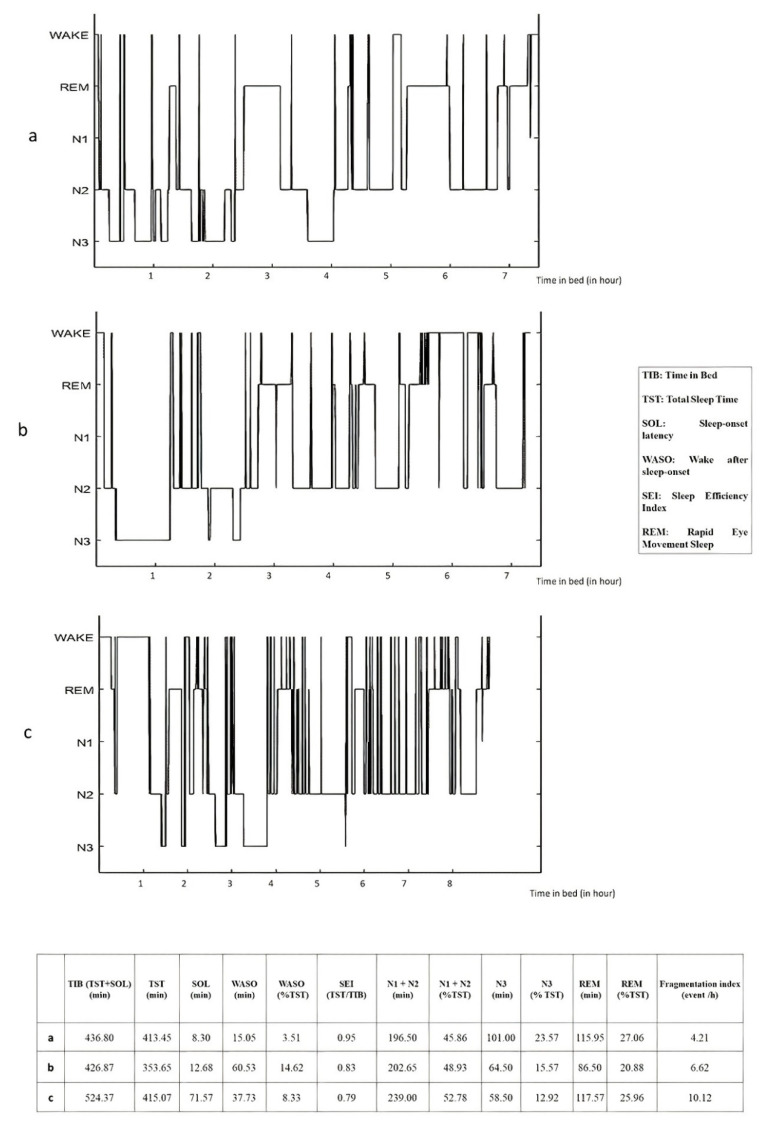
Hypnograms for one French soldier (35 years old) before (**a**) and after (**b**) deployment and one soldier with PTSD (36 years old) (**c**). Night sleep EEG recordings were performed using the ambulatory wireless dry-EEG device, the Dreem Headband (DH), with automatic sleep staging classification (122). Fragmentation index is the total number of awakenings/shifts to Stage 1 (from deeper non-rapid eye movement [NREM] or REM sleep with 30 s per epoch) divided by the total sleep time in hour. Other parameters are considered to reflect sleep fragmentation, such as increased WASO or a high percentage of the N1/N2 sleep stage, which usually results from frequent arousal. The figure shows that the veteran without PTSD has good sleep efficiency and a low fragmentation index prior to deployment. During deployment, there is an increase in fragmentation index and WASO associated with a decrease in sleep efficiency and N3 sleep. The veteran with PTSD has low sleep efficiency with a high fragmentation index. The percentage of REM is high while that of N3 is rather low. Unpublished data from ongoing clinical trial SOMMEPT (https://clinicaltrials.gov/ct2/show/NCT04581850) (accessed on 10 June 2021).

Nightmares are a specificity of this disorder. Trauma-related nightmares (TRN) are the most common symptom of PTSD, highly de-stressing and associated with severity, treatment resistance and chronicity [53]. TRN are highly associated with deployments and combat-related events [54]. In most cases, TRN are replicative (an exact replication of the traumatic event) and associated with a sudden awakening, tachycardia, diaphoresis and intense anxiety [55]. In addition, some external factors such as treatment with the antimalarial drug mefloquine may play a role in the occurrence of nightmares, but these nightmares are not formally related to deployment experiences [56,57,58], and thus should be differentiated from traumatic nightmares. The study of TRN is difficult because the location and conditions of recording can influence the frequency and content [59,60]. However, studies tend to show that TRN occur in both REM and non-REM sleep [3,60]. In addition, TRN have some characteristics of classic REM nightmares (accurate storyline, elements of reality, and full awakenings) but also characteristics of non-REM sleep (i.e., they occur early in the sleep period and are accompanied by anxiety and large body movements) [3,61,62]. Furthermore, it would be very interesting to differentiate TRN from classic nightmares because they are a typical sign of the disorder and probably of its evolution [62,63]. That’s why some experts consider sleep disturbances and TRN as a hallmark of PTSD [1,62]. However, it remains extremely difficult to corroborate these subjective complaints with laboratory objective sleep measures [4]. The sleep laboratory, often perceived as a safe environment, may promote sleep, which is a bias. Some studies have attempted to take ambulatory sleep measurements at home, but the size and bulk of the recording devices prevent repeat measurements and, despite all precautions, alter the environment and limit the results [64]. Results of a recent review and meta-analysis of 31/34 appropriate polysomnographic studies described disturbances in sleep architecture and sleep continuity in patients with PTSD compared with healthy controls [65]. Overall, PTSD is associated with low sleep efficiency (Total sleep time/Time in bed), a longer sleep latency, increased wake after sleep onset (WASO), and a shorter total sleep time [63,64,65]. When compared to healthy controls, the SWS percentage is also found to be lower [65]. In addition, sleep is more fragmented with more nocturnal awakenings and increased arousal [1,2,66]. With regard to the sleep architecture in PTSD, the results are not consistent, with some studies finding an increase in the proportion of REM sleep and others finding a decrease [3,4,67]. Examples of these findings are shown in Figure 1. In a recent study, Rousseau et al. investigated sleep parameters before and after EMDR (Eye movement desensitization and reprocessing) therapy in French soldiers with PTSD. Interestingly, it appears that there is an increase of REM sleep quantity and density after symptom remission [68].

Recent data show there is a need to further study sleep microstructure in PTSD. In particular, spectral power analysis could be a promising biomarker in this disorder [69], but spindle oscillatory frequencies and phase synchronies in specific bands show promise as well [69,70].

These characteristics lead some authors to mention trauma-associated sleep disorder (TSD) as a distinct parasomnia that is not necessarily a comorbidity of PTSD [62]. TSD appears to be more prevalent in cases of combat-related PTSD, especially because sleep deprivation and/or disruption during deployment may be a precipitating factor [62]. TSD is described, after combat or other extreme traumatic experience, by (1) altered dream mentation; (2) disruptive nocturnal behaviors (screaming or motor behaviors); (3) autonomic hyperarousal during sleep; and (4) REM sleep without atonia or dream enactment behavior during REM sleep as seen on PSG recordings [62,71]. This definition of TSD is very interesting because, within the broad category of PTSD, it identifies a subgroup of patients (particularly veterans with combat-related PTSD) for whom sleep may be of particular importance both in the physiology of the disorder and its clinical manifestations.

A majority of patients with chronic PTSD and nightmares do not receive optimal treatment targeting nocturnal symptoms [72]. However, psychological treatments are effective for treating PTSD, anxiety, and depression and improving sleep in people (including military personnel) with a history of complex traumatic events. Pharmacological interventions are less effective than psychological interventions for treating PTSD symptoms and improving sleep, with a high risk of side effects [73]. At present, even if the improvement in sleep is correlated with the overall improvement in PTSD, few pharmacological or psychotherapeutic treatments showed a significant effect on sleep, probably because there are currently no clinical guidelines for screening sleep disorders in PTSD [74]. That’s why sleep disturbances remain a frequent residual complaint after successful treatment of PTSD [60,75].

#### 3.2.2. Dynamic Link between Sleep and the Emergence of Military PTSD

It is very interesting to note the association between the intensity of sleep disturbances and PTSD daytime symptom severity. Indeed, TRNs are independently associated with daytime distress and dysfunction [76]. In a study, insomnia was the most commonly reported symptom of PTSD and predicted the other symptom clusters of PTSD in a group of war veterans [77]. Reciprocally, improvements in nightmares and insomnia are accompanied by an improvement in the severity of daytime symptoms of PTSD, depression and anxiety [3,5,78]. In PTSD, sleep appears to be a mediating symptom that exacerbates PTSD (intensity and chronicity), which is why some authors argue that sleep-focused treatment should be routinely used early in the treatment of PTSD. Additionally, some research suggests that specific psychotherapies targeting sleep could be efficient in veterans, reducing nightmare frequency and nightmare distress, and improved sleep quality [79]. This is consistent with the DSM-5 guidelines: sleep disorders should be diagnosed and treated even if they are comorbid with another mental disorder [78].

Many studies support the hypothesis of a dynamic role of sleep in the constitution of PTSD. Indeed, the presence of nightmares or sleep disturbances before the deployment is associated with an increased risk of post-deployment PTSD [36,80,81]. On the other hand, acute post-traumatic insomnia can become chronic and disrupt processes of sleep-dependent emotional memory consolidation, thereby contributing to the aetiology of PTSD [82]. The presence of nightmares and sleep disturbances after a traumatic event has been shown to be predictive of the onset of PTSD [80,81,83], and sleep fragmentation predicts TSD [60]. The REM segment duration during the early aftermath of trauma is also correlated negatively with initial PTSD and insomnia severity [84].

PTSD is also found to be strongly associated with sleep-disordered breathing (in 67–69% of veteran populations) [85]. The explanation for this association remains unclear and there is probably a bi-directional pathway. Indeed, soldiers may have suffered from pre-existing undiagnosed OSA, which is responsible for sleep fragmentation and increased susceptibility to developing PTSD. But PTSD could also aggravate sleep-related sub-clinical respiratory disorders [86]. In addition, another stressor for the military, such as the use of malaria prophylaxis, could potentiate or even cause OSA, due to its mechanistic action toward a loss of tone in the genioglossus [87]. The gold standard OSA treatment, positive airway pressure therapy, seems to decrease insomnia, nightmares and post-traumatic stress symptoms in patients with PTSD [86].

The amount of time that has passed since the trauma is probably an important mediating factor. In fact, if a majority of studies focus on the chronic phase of PTSD (when patients with this disorder are clearly identified), there is a lack of objective data on sleep in the days directly following the traumatic event up to chronic PTSD. Recent findings show insomnia as an independent risk factor for the worsening of PTSD [86,88].

Overall, although the significance of the statistical association is not yet fully elucidated, there is significant evidence showing a strong link between sleep and PTSD. It is very likely that sleep represents a transition factor from trauma to PTSD. As trauma appears, by definition, to be an unpredictable event, care strategies targeting sleep should aim to prevent the transition to chronic PTSD.

## 4. A Reciprocal Relationship

### 4.1. The Reciprocal Relationship Model: A Reality

**Figure 2:**
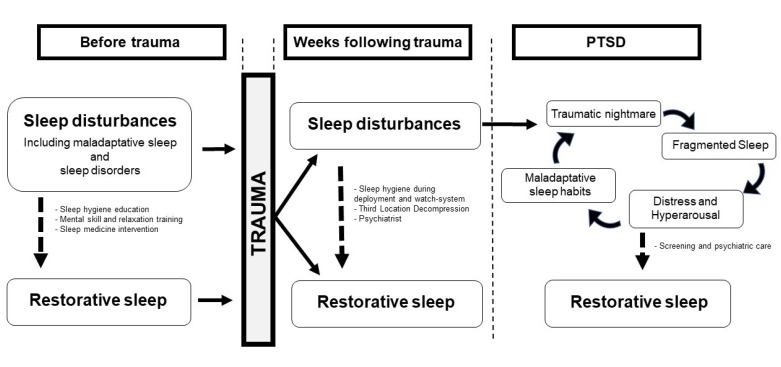
Sleep disturbances, PTSD and specific interventions in military.

Altered sleep quality before and also after the trauma seems to be a mediating factor of PTSD (Figure 2). The clinical logic is therefore to prevent and reduce sleep disturbances in order to promote restorative sleep. The military population has similar sleep disorders as the general population, but also maladaptive sleep (sleep restriction and/or fragmentation) due to the military operational context with its specific constraints [8]. Military missions on home territory and training are pre-deployment conditions that can induce inadequate sleep [9]. This is why the French army’s health services have developed an intervention model to detect, prevent and treat sleep disorders. Prior to deployment, all soldiers receive sleep hygiene education sessions, with the goal of providing benchmarks by informing them of the benefits of maintaining good quality sleep and educating them on sleep management [89]. Moreover, all soldiers are sensitized to mental skills and relaxation training. This is done through a set of techniques called relaxation techniques (RTs), which include positive reinforcement, mental imagery, mental revitalization, and muscle relaxation [90]. These techniques allow soldiers to relax, particularly in the evening or before naps. The challenge is to be able to fall asleep easily and to limit sleep fragmentation due to stress. The RTs program has been shown to be effective in promoting sleep in the workplace [91].

In the French army, the state of health of military personnel is monitored regularly [92]. Before deployment, all soldiers benefit from a medical consultation. In addition to discussing personal and professional factors, physicians screen for sleep disorders and, if necessary, offer specialized care prior to deployment. The challenge is to maintain restful sleep throughout the deployment. We are also careful to limit stressors liable to degrade sleep (as is the case with antimalarials such as mefloquine, which is no longer used in the French army) [57]. To this end, the staff who supervise and command are made aware of the management of sleep rhythms and a wakefulness system is generally set up. If there is a traumatic event, the affected soldier benefits from defusing, generally debriefing and if necessary, consulting with a military psychiatrist directly during deployment. In addition to verbalization effects, some drugs can be used to promote sleep. They include antihistaminic (e.g., HYDROXYZINE 25 mg before sleep for a few days) and a short acting, atypical antipsychotic in low doses (e.g., LOXAPINE 20 mg before sleep for a few days). If psychotraumatic symptoms persist over time, the patient is medically repatriated to a military hospital in France for specialized care. Even in the absence of a traumatic event and/or symptoms, soldiers benefit from a third location decompression (TLD) program between deployment and return to France. Many other countries, including Canada, the Netherlands, Australia, and the UK, have established TLD programs for their service members [93,94]. Countries that employ TLD programs for their service members often choose sites with high-quality lodging and ample recreational activities (tourist waterfront or sightseeing locations) (i.e., Cyprus). The French TLD program has been described previously: the activities are varied and set up over three days to facilitate the transition from deployment area [95]. During these days, soldiers can relax and participate in various information workshops on RTs, sleep, and psychotraumatic risk. In the course of the TLD, a new screening for sleep disorders and psychotraumatic symptomatology is performed. Patients with significant symptoms are referred to a consultation with a military psychiatrist. In the weeks following the return, a new medical-psychological evaluation is conducted for screening purposes.

In addition to the link between sleep disorders and the onset of PTSD, there is a second level of entanglement when PTSD symptoms become chronic. Indeed, PTSD itself generates sleep disturbances through TRNs. Whether or not they are the hallmark of PTSD, TRNs allow a vicious circle to develop. In this respect, sleep can be considered an aggravating factor for PTSD.

TRNs induce intrusive phenomena of the traumatic experience that maintain a state of hypervigilance and stress. Abrupt awakenings lead to significant sleep fragmentation that generates chronic asthenia and also maintains irritability and cognitive disorders. In addition, PTSD patients develop poor sleep habits because of the fear of sleep. In general, a very late bedtime (after midnight) with sleep avoidance behavior (late use of screens) is observed. The fear of not being able to fall asleep can lead to the use of a disordered substance: cannabis or alcohol. There is a very strong comorbidity between alcohol use disorders and PTSD [96]. Combat-related trauma, in particular, has been linked to significant increases in heavy alcohol use post-deployment, when compared to alcohol use pre-deployment [97]. Alcohol consumption is often described as heavy evening drinking for anxiolytic and hypnotic purposes. This consumption is also likely to promote the development of OSA, which in turn will further degrade sleep.

The request for specialized care is often due to a complaint about sleep. This is one of the first requests for care from patients for which we have very few solutions. With respect to sleep interventions, attention has turned to the role of multidisciplinary and integrative approaches as comprehensive treatment for sleep disturbances in PTSD [98].

### 4.2. Physiological Hypotheses

Regarding our model on the interrelationship between sleep disorders and PTSD (Figure 2), we hypothesized that sleep fragmentation, which is a feature identified before the trauma and in the weeks following the trauma, is a predictor of chronic PTSD.

Sleep fragmentation has been defined as an interruption (disruption) of the normal continuity of sleep with frequent (as often as l per minute) and transient (3–10 s) electroencephalogram (EEG) arousals [99]. Sleep fragmentation is generally estimated with the sleep fragmentation index (SFI), which is the total number of awakenings/shifts to Stage 1 (from deeper non-rapid eye movement [NREM] or REM sleep) divided by the total sleep time in hours [100].

Arousals are observed in various sleep disorders and can be identified in some specific conditions (i.e., apnea, leg movements, pain). The arousals produce fragmented rather than shortened sleep, and the fragmented sleep is associated with impairment of daytime function [101]. This is one of the reasons why there are patients who complain about their sleep when it is objected that they have a sleep macrostructure that appears to be minimally disturbed but with a significant SFI [66]. Before and during military deployment, many factors can increase sleep fragmentation and thus contribute to a lack of restorative sleep [11]. Sleep fragmentation may lead to common symptoms including increased objective sleepiness, fatigue, and decreased psychomotor performance on a number of tasks, including tasks involving short term memory, reaction time, vigilance, and degraded mood [102]. Activation of the sympathetic nervous system, the sympathoadrenal system, and the hypothalamic–pituitary–adrenal axis are involved in the consequences of sleep fragmentation [102]. There may be a positive feedback link between central stress systems, sleep and REM sleep disruption, and altered extinction memory as a possible pathway to worsening post-traumatic psychopathology [82]. Thus, sleep fragmentation would exert negative effects on recovery from trauma and combat-related physiological consequences. Sleep fragmentation also exerts an adverse effect on pain sensitivity in young healthy adults [103] and is bi-directionally linked with alcohol misuse [104]. Moreover, sleep fragmentation represents the common thread in the relationship between PTSD and sleep-disordered breathing [105]. Long-term consequences of sleep disruption in otherwise healthy individuals include hypertension, dyslipidaemia, cardiovascular disease, weight-related issues, metabolic syndrome, type 2 diabetes mellitus, and colorectal cancer [106].

With respect to PTSD, it has been described that REM sleep fragmentation in the early aftermath of trauma predicts the development of PTSD symptoms [66], suggesting a causal relationship between REM disruption and PTSD onset. To date, no studies have examined SWS disruption following trauma, although it has been postulated that there is a sequential and complementary role of NREM sleep (stabilizing a memory trace) followed by REM sleep (integrating the traumatic experience by reducing affective tone) in trauma memory formation [107].

PTSD has been shown to be marked by SWS and REM disruptions at both the macro and micro levels. Kobayashi et al. reported that PTSD was associated with a reduction in PSG sleep continuity as compared with controls [108]. Intra-sleep awakenings in PTSD before beginning an EMDR treatment are correlated with the number of treatment sessions required to reach remission [68]. In addition, TRN have been shown to occur at different stages of sleep and are not limited to REM sleep [109], and to induce more motor activity during NREM and REM sleep stages [3].

In a study, patients with PTSD had significantly higher awakening thresholds during sleep stages 3 and 4 [110], suggesting an important role of NREM sleep continuity in PTSD. Furthermore, the duration of the NREM is described as reduced in patients with PTSD compared to healthy controls, and the severity of PTSD is associated with the percentage of SWS [65]. Concerning REM sleep, disruption or fragmentation of REM sleep was reported in PTSD and/or trauma survivors compared with controls, including PTSD-positive vs. PTSD-negative veterans, and PTSD-positive vs. normal healthy civilians [111]. REM sleep disturbances were observed in PTSD combat-exposed military veterans (aged between 37 and 49 years) compared to an age-appropriated normal control group: the REM sleep percentage, average REM activity and average REM period duration were significantly higher [112,113]. In the recent Zhang et al. [65] meta-analysis from 31 studies, the REM sleep percentage is significantly lower in PTSD patients compared with controls in studies where the mean age of participants was below 30 y, but not in studies with greater mean ages (30–40 years and >40 years). These differences of REM sleep percentages in PTSD may reflect an adaptive process in chronic patients, as suggested by Mellman [114]. This hypothesis is supported by a recent study that suggests that increased REM sleep density should be a possible marker of treatment success [68].

REM sleep disturbance subsequent to trauma exposure was found to be associated with dysfunction in emotional memory processing, increased trauma-related nightmares in PTSD, and impairments of conditioned fear extinction [78,113]. A recent study demonstrated that REM fragmentation in PTSD, and not slow-wave sleep, predicts neutral declarative memory consolidation [100].

In conclusion, there is a body of evidence that sleep fragmentation (of the two stages, REM and slow-wave sleep) impacts the overall sleep architecture and contributes to the development, maintenance, and severity of PTSD. There could be a mechanistic impact of REM and NREM sleep fragmentation in the processing of traumatic memories, but this requires further studies to be elucidated.

## 5. Clinical and Research Perspectives

From a clinical point of view, considering sleep fragmentation in the military population seems fundamental. In the area of prevention, measures that have been put in place (naps, shift systems, screening) must be continued and improved upon. This requires that leaders be made aware of these data [8], and that psychiatrists be more aware of the interrelationship between sleep disturbances and PTSD in order to perform or have performed a PSG to objectify and manage sleep disturbances.

In the therapeutic field, different perspectives seem promising. It should be noted that drug therapies targeting traumatic nightmare (such as prazosin) have been disappointing [115]. Conventional drug treatment of PTSD has opposite effects on sleep fragmentation: SSRIs are associated with greater sleep fragmentation and antipsychotics are known to moderately improve sleep continuity [64]. The effects of these treatments on sleep in PTSD should be further studied to make prescriptions as individualized as possible.

Mechanical and respiratory approaches may be relevant to restore sleep continuity. For the treatment of OSA comorbid with PTSD (severely underdiagnosed in the military), CPAP therapy is the gold standard, reducing not only the symptoms of sleep apnea but also those of PTSD, including anxiety, depression, nightmares and quality of life [105]. In military personnel diagnosed with insomnia or comorbid insomnia with OSA receiving four to eight biweekly sessions of cognitive behavioral therapy for insomnia (CBT) and CPAP for their OSA, improvements in sleep quality were associated with significant declines in depression and posttraumatic arousal symptoms, and a significant increase in plasma concentrations of IGF-1, the growth hormone dependent factor implicated in memory processes [7,116]. This may be of particular interest because Van Liempt et al. [117] found decreased nocturnal GH secretion and sleep fragmentation in combat-related PTSD disorder. The major problem with CPAP treatment is compliance due to mask discomfort, claustrophobia and air hunger [118]. Therefore, mandibular advancement devices, which have also been shown to be effective in reducing PTSD symptomatology, may be a better tolerated alternative [105].

From another perspective, in order to re-establish good sleep hygiene habits, the development and implementation of therapeutic education modules focused on sleep and adapted to soldiers suffering from PTSD should be encouraged. Interestingly, a recent study in adults with PTSD and insomnia (aged a mean of 37 years) demonstrated that eight weeks of cognitive behavioral therapy for insomnia (CBT-I) not only improves insomnia symptoms but also may improve PTSD symptoms, including fear of sleep [119].

A significant amount of current research is devoted to identifying biomarkers of PTSD, including sleep and REM sleep disruption, ideally through an ecological home survey [120]. For this, many studies concern the development of connected devices that would measure sleep at home [121,122]. Obtaining biomarkers related to sleep fragmentation, such as a traumatic nightmare pattern, could allow for real-time manipulation of sleep to limit nighttime awakenings [123]. This is why it is necessary to develop machine learning tools to identify specific patterns combining different variables (EEG, actimetry, heart rate, electrodermal conductivity, etc.) that are potentially invisible to the human eye and independent of the steps described conventionally [124].

## 6. Conclusions and Implications for Non-Military PTSD Disorders

Sleep disturbances are an ongoing challenge for clinicians who care for psychologically and/or physically traumatized individuals. This review examined alterations in a variety of sleep architectures and sleep patterns described during military deployment and also in combat-related PTSD. Evidence from the literature underscores the importance of considering sleep fragmentation as a factor in the transition from trauma to chronic PTSD. The dynamic study of sleep fragmentation could be relevant to examine the relationship between deployment-related sleep stressors, sleep-disordered breathing, and traumatic nightmares.

We discussed clinical and research perspectives that could be used to detect or restore sleep continuity before and during military deployment to potentially alleviate nightmares and insomnia related to combat exposure and the onset of PTSD and to improve our understanding of sleep in PTSD. There is a need to develop devices to record sleep in an ecological way. Indeed, more data are needed to be able to understand precisely the causal relationship between sleep fragmentation and the chronicization of PTSD.
